# Reduction in Radiation Exposure through a Stress Test Algorithm in an Emergency Department Observation Unit

**DOI:** 10.5811/westjem.2015.12.27895

**Published:** 2016-03-02

**Authors:** Margarita E. Pena, Michael R. Jakob, Gerald I. Cohen, Charlene B. Irvin, Nastaran Solano, Ashley R. Bowerman, Susan M. Szpunar, Mason K. Dixon

**Affiliations:** St. John Hospital and Medical Center, Department of Emergency Medicine, Detroit, Michigan

## Abstract

**Introduction:**

Clinicians are urged to decrease radiation exposure from unnecessary medical procedures. Many emergency department (ED) patients placed in an observation unit (EDOU) do not require chest pain evaluation with a nuclear stress test (NucST). We sought to implement a simple ST algorithm that favors non-nuclear stress test (Non-NucST) options to evaluate the effect of the algorithm on the proportion of patients exposed to radiation by comparing use of NucST versus Non-NucST pre- and post-algorithm.

**Methods:**

An ST algorithm was introduced favoring Non-NucST and limiting NucST to a subset of EDOU patients in October 2008. We analyzed aggregate data before (Jan-Sept 2008, period 1) and after (Jan-Sept 2009 and Jan-Sept 2010, periods 2 and 3 respectively) algorithm introduction. A random sample of 240 EDOU patients from each period was used to compare 30-day major adverse cardiac events (MACE). We calculated confidence intervals for proportions or the difference between two proportions.

**Results:**

A total of 5,047 STs were performed from Jan-Sept 2008–2010. NucST in the EDOU decreased after algorithm introduction from period 1 to 2 (40.7%, 95% CI [38.3–43.1] vs. 22.1%, 95% CI [20.1–24.1]), and remained at 22.1%, 95% CI [20.3–24.0] in period 3. There was no difference in 30-day MACE rates before and after algorithm use (0.1% for period 1 and 3, 0% for period 2).

**Conclusion:**

Use of a simple ST algorithm that favors non-NucST options decreases the proportion of EDOU chest pain patients exposed to radiation exposure from ST almost 50% by limiting NucST to a subset of patients, without a change in 30-day MACE.

## INTRODUCTION

There has been increased medical and public awareness regarding radiation exposure in medical procedures. The U.S. Food and Drug Administration recently issued an initiative to reduce unnecessary radiation from medical imaging and to encourage physicians to order the appropriate diagnostic test for the appropriate patient and only when medically justified.^1^ Guidelines from the American College of Cardiology (ACC) and the American Heart Association (AHA) as well as appropriate-use criteria from the ACC Foundation Quality Strategic Directions Committee Appropriateness Criteria Working Group and the American Society of Nuclear Cardiology have been published that aim at reducing inappropriate use of single photon emission computed tomography myocardial perfusion imaging (SPECT-MPI) as an initial test in low and intermediate risk patients.^2^,^3^ Unfortunately, despite these recommendations and although there have been methods introduced to decrease radiation doses for nuclear stress tests (NucST), a 2011 member survey of the American Society of Nuclear Cardiology reported that no significant assimilation of these approaches into clinical practice had occurred.^4^

In the U.S., approximately 10 million NucST are ordered per year.^4^ The amount of radiation from a NucST using technetium (99Tc) tetrofosmin is 11.4mSv, which is equivalent to about 570 portable single-view or 114 two-view chest radiographs.^5^ The cancer risk projection of having this NucST at age 50 years is estimated to result in a lifetime risk of 10 cancers per 10,000 tests and is increased to 25 cancers per 10,000 tests for dual isotope (thallium-201 plus technetium-99m) NucST.^6^

Furthermore, this cancer risk is not static, as in younger patients this projection ratio increases.^6^ Given that 10 million tests are performed per year, and given the known cancer risk (10 per 10,000 for 50-year-olds), an algorithm that decreased the number of NucST by even 20% would result in substantially less patients with risk of cancer (potentially estimated 2,000 patients with no increased cancer risk if all were 50 years old).

Stress testing is an important diagnostic tool in evaluating emergency department (ED) patients who present with chest pain (CP). Practice guidelines and algorithms for choosing a stress test based on risk stratification, ability to exercise and clinical features, have been published.^7^–^10^ While there is mention of radiation exposure consideration when choosing one ST option over another, to date there is no study that has estimated the magnitude of decreased radiation exposure after adoption of a ST algorithm that favors non-NucST options. The objective of this study was to introduce a simplified ST algorithm for low-risk CP patients placed in an emergency department observation unit (EDOU) that favors non-NucST options and assess the impact on the proportion of NucST vs Non-NucST performed, then determine if adoption of this algorithm would have an impact on 30-day major adverse cardiac events (MACE). A secondary objective was to evaluate algorithm adherence by emergency physicians.

## METHODS

### Study Design

This was a retrospective observational study of EDOU patients who underwent stress testing between January 2008 and September 2010 in an urban teaching hospital with >100,000 ED patient visits per year. The EDOU is a 30-bed unit located one floor directly above the ED that is managed and staffed by emergency physicians. This study was approved by the hospital institutional review board.

### Study Setting and Population

Our EDOU inclusion criteria for stress testing included adult ED patients with a concern for acute cardiac syndrome (ACS) who were low risk for ACS as defined by the ACC/AHA in 2007 and included patients with an initial normal or non-diagnostic electrocardiogram (ECG) and an initial normal cardiac troponin T level.^11^ All patients had a repeat ECG and troponin T performed after four hours in the EDOU. Only patients with a repeat unchanged ECG and negative second troponin T level were candidates for stress testing. Our hospital uses the Roche Diagnostics assay Troponin T, cardiac T (cTnT) measured in our central laboratory with a lower limit of detection of 0.01mcg/L and a 10% coefficient of variation of 0.03–0.06mcg/L. A decision limit for normal of <0.03mcg/L and a positive value as ≥0.10mcg/L is used.

### Study Protocol

**Figure 1:**
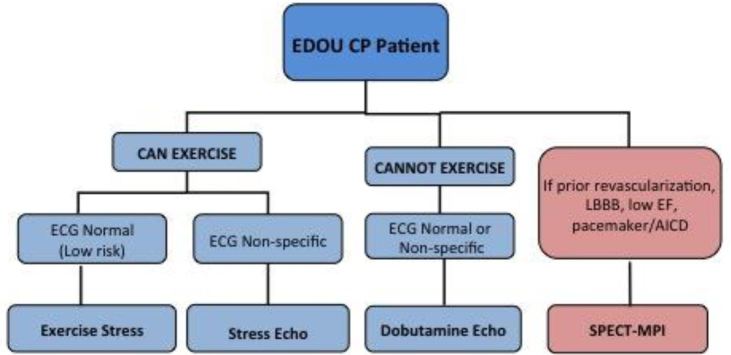
A simple stress test algorithm for patients in the emergency department observation unit (EDOU). *CP*, chest pain; *ECG*, electrocardiogram; *LBBB*, left bundle branch block; *EF*, ejection fraction; *AICD*, automatic implantable cardioverter-defibrillator; *SPECT-MPI*, single photon emission computed tomography myocardial perfusion imaging

As part of a process improvement project, physicians from the departments of emergency medicine and cardiology collaborated using evidence-based literature to construct an algorithm in October 2008 that favored Non-NucST over NucST and limited nuclear imaging only to patients with known low ejection fraction (<50%), history of coronary revascularization, pacemaker or AICD, or left bundle branch block (Figure 1). Emergency physician education of the algorithm was implemented in October 2008 and was reinforced intermittently thereafter. Physician (attending and resident) education included introduction and explanation of the algorithm and lectures related to ED CP evaluation, risk stratification and stress testing. The algorithm was also posted in the physician workspaces.

### Stress Testing

Non-NucST included the graded exercise stress test (GXT), stress echocardiography (SE) and dobutamine echocardiography (DE). GXTs were performed according to AHA/ACC guidelines using a Bruce protocol, with increased treadmill speed and velocity every three minutes, continuous symptom and ECG monitoring, and termination according to ACC/AHA guidelines.^7^ The patient recovered from exercise by walking slowly on the treadmill until heart rate was less than 100bpm or by resting in a supine or seated position if unable to walk in recovery. For SE, the patient transferred as quickly as possible from the treadmill after peak exercise to the left decubitus position for imaging. The heart rate recovery, or heart rate one minute post exercise, was recorded. Ischemia was noted if at least 1mm flat or down-sloping ST depression was present on ECG, a new or worsened segmental wall motion abnormality was detected on echocardiography, or if a new or worsened perfusion defect was present on nuclear imaging. When the endocardium was not adequately visualized during echocardiography, intravenous contrast (Optison [GE Healthcare, Milwaukee WI] or Definity [Lantheus, N. Billerica, MA]) was infused.

Pharmacologic stress testing was performed using intravenous dobutamine primarily combined with echocardiography, or adenosine or regadenoson combined with nuclear imaging. Graded dobutamine infusion was performed by increasing infusion dosage every three minutes by 10ug/kg/min increments, to a maximum of 50ug/kg/min, with a targeted heart rate of 85% of the age-predicted maximum. The infusion was supplemented by intravenous atropine if heart rate response to dobutamine was inadequate. Four standard images were obtained at rest, 10 ug/kg/minute infusion, peak infusion, and recovery (when heart rate had dropped to less than 100bpm). Intravenous metoprolol (or diltiazem if metoprolol was contraindicated) was administered in most patients to terminate the effects of dobutamine during recovery. NucST was performed with single photon emission computed tomography myocardial perfusion imaging (SPECT-MPI) after tetrofosmin injection and at two to four hours of rest after the first injection.

### Measurements

We compared aggregate data of stress test utilization from patients presenting to the EDOU during three similar time periods: period 1 (baseline phase: January–September 2008), period 2 (intervention phase: January–September 2009), and period 3 (maintenance phase: January–September 2010). The algorithm was introduced in October 2008 after period 1 and before period 2.

To assess for adherence to the algorithm, the department of cardiology kept a log of all ST that required re-ordering due to incorrect test selection after introduction of the algorithm (periods 2 and 3). These data were used to obtain the percent of correct ST ordered (i.e. the ST ordered by the ED physician did not need to be changed to another type) and were used as a surrogate for algorithm adherence. We compared Period 2 to Period 3 to assess retention.

We used a random sample of 240 patients from each period to assess for 30-day MACE before and after algorithm implementation. Data were collected using the hospital electronic medical record and the National Death Index. Data collection included demographics (age, gender, race); presence of cardiac risk factors defined as hypertension, diabetes, hyperlipidemia, family history, and tobacco use; and history of cardiac co-morbidities defined as coronary artery disease, myocardial infarction, percutaneous intervention or coronary bypass artery grafting. We defined MACE as acute coronary syndrome (ACS), catheterization with lesions >50% requiring percutaneous coronary intervention (PCI), coronary artery bypass grafting (CABG), and death.

### Data Analysis

Descriptive statistics were generated to characterize the study population. Data are presented as means or proportions with 95% confidence intervals. We calculated confidence intervals for proportions or the difference between two proportions without the continuity correction using the method of Wilson.^12^ Data was analyzed with SPSS v. 22.0.

## RESULTS

A total of 5,047 EDOU patients underwent ST during the three time periods: 1,584 in Period 1, 1,645 in Period 2, and 1,867 in Period 3. Of the 720 patients randomly selected during the first three time periods, six pwere excluded due to missing or incomplete data.

NucST in the EDOU decreased from Period 1 to 2 (40.7%, 95% CI [38.3%–43.1%] vs. 22.1%, 95% CI [20.1%–24.1%]), and remained at 22.1%, 95% CI [20.3%–24.0%] in Period 3. See Table 1. The mean proportion of correct stress tests ordered by emergency physicians/residents was similar between Period 2 (91.3%, 95% CI [83.8–95.2]) and Period 3 (89.6%, 95% CI [81.4–93.8]).

**Figure 2:**
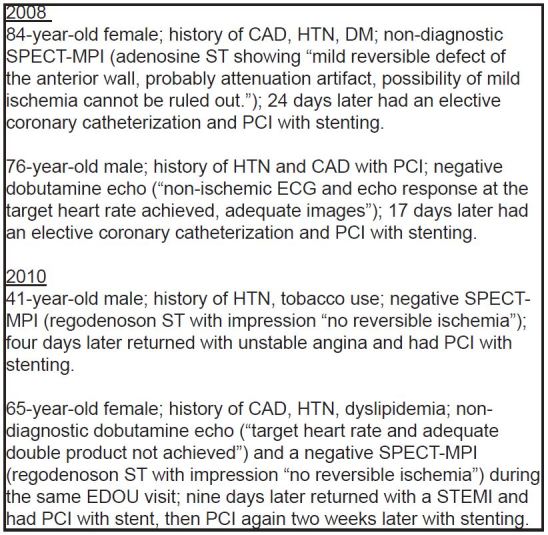
Patients with a major adverse cardiac event within 30 days of emergency department observation unit (EDOU) visit. *CAD*, coronary artery disease; *HTN*, hypertension; *DM*, diabetes mellitus; *SPECT-MPI*, single photon emission computed tomography myocardial perfusion imaging; *PCI*, percutaneous coronary intervention; *ECG,* electrocardiogram; *STEMI*, ST elevation myocardial infarction; *ST*, stress test

In the random sample of 714 patients during Periods 1, 2 and 3, there were no significant differences in mean age, distribution by race, or history of cardiac co-morbidities. See Table 2. There was a difference between periods by gender, with 32.23% male in Period 1 (95% CI [26.6–38.4]), 30.2% in Period 2 (95% CI [24.7–36.4]) and 41% in Period 3 (95% CI [35.0–47.3]). There was no difference in MACE within 30 days of index EDOU visit between periods. Two patients in Period 1 (0.1%) returned within 30 days of their index visit and underwent PCI, and two patients in Period 3 (0.1%) returned and were found to have ACS and subsequently required PCI during that hospital visit. No patients in Period 2 returned to the hospital with MACE. Figure 2 has the details of the four patients with a 30-day MACE.

## DISCUSSION

It is important to find ways to decrease radiation exposure to our patients. In this study, introduction of a simple ST algorithm that promotes Non-NucST options and limits NucST in EDOU CP patients decreased NucST utilization by almost 50%. This decrease in utilization of NucST was sustained over two years. One factor that may have contributed to this is that the algorithm is specific in limiting NucST only to the subset of patients with certain cardiac co-morbidities where nuclear testing is optimal. All other patients regardless of cardiac risk factors or other cardiac co-morbidities, such as history of coronary artery disease without revascularization or diastolic heart failure with preserved ejection fraction, are eligible for Non-NucST options. The algorithm included ST options that were consistent with current guidelines (ACC/AHA) and previously published algorithms.^7^–^10^ However, it was important for our process improvement goal of decreasing radiation exposure to our EDOU CP patients that the algorithm be both specific in limiting NucST only to a subset of patients with certain cardiac co-morbidities where nuclear testing is optimal and be easy to use by our attending and resident staff. Although it is intuitive that radiation exposure will be less if non-NucST is used and preferred over NucST, this is the first study to attempt to quantify the decrease in radiation exposure after the algorithm initiation.

Although NucST does have higher sensitivity than Non-NucST (85% vs. 79%)^13^ and has the advantage of being able to identify distinct lesion sites,^14^ for most low-risk CP patients requiring stress testing in an EDOU setting, this higher sensitivity is not needed. Because the negative predictive values for ACSs and death are similar Non-NucST and NucST (96.6%–98.8% vs. 97.4%–98.4%), either could be used to safely discharge a patient if negative.^15^ The findings of our study support this premise; in our subset analysis of the 714 randomly selected patients, there was no difference in 30-day rates of MACE before or after use of the ST algorithm. In the study by Buchsbaum et al of low cardiac risk patients who presented to the ED with CP, of the 138 who had normal stress echocardiograms, all were cardiac event free at three-month follow up and only one had a cardiac event at six months.^16^ This is similar to what we found in our study.

Emergency physicians correctly ordered the appropriate stress test approximately 90% of the time suggesting the algorithm is easy to use and assimilate into practice. Therefore, this simple ST algorithm seems to be practical for clinical use in assigning which ST to choose for patients who are placed in an EDOU setting for evaluation of CP.

The AHA and the ACC guidelines for appropriate use of NucST imaging in the evaluation of CP suggest that NucST imaging in CP evaluation should be reserved for a distinct set of high-risk patients.^2^,^3^ The importance of appropriate ST selection is re-emphasized, as patients with a longer life expectancy will also be at increased risk of cancer directly related to nuclear imaging.^6^ Recently, Eisenberg et al. stated that patients exposed to low-dose ionizing radiation from cardiac imaging are unequivocally at an increased risk of cancer,^17^ lending support in favor of Non-NucST when possible. Furthermore, use of Non-NucST options for evaluation of CP specifically in women (the majority in our study) is also supported by the AHA.^18^ The necessity and utility of implementing such algorithms and guidelines is therefore clearly indicated and can be argued that it is also of some urgency. The U.S. radiation burden from nuclear cardiology increased from 1% of all radiation exposure to patients in 1982 to 10.5% of total radiation exposure in Americans in 2006. Clearly the increase in use of NucSt is substantial and the effects of such radiation to the population are unequivocally not without harm.^6^,^19^

In the U.S., approximately 10 million NucST are ordered per year.^4^ The amount of radiation from a NucST using technetium (99Tc) tetrofosmin is 11.4mSv, which is equivalent to about 570 portable single view or 114 two-view chest radiographs.^5^ The cancer risk projection of having this NucST at age 50 is estimated to result in a lifetime risk of 10 cancers per 10,000 tests.^6^

Given the 18.6% absolute risk reduction (40.7% reduced to 22.1%) of NucST after the new algorithm, the five-year estimated lifetime risk of cancer changed (assuming all were age 50) from 4.3 patients to 2.3 patients at this institution alone. To understand the potential national effect, extrapolating to the 10 million NucST/year (assuming the pre-algorithm rate of NucST was similar and assuming all were 50 years old), the 18.6% reduction equates to an estimated reduction in lifetime risk of cancer for 1,860 patients/year after institution of the algorithm. While extrapolation nationally may not be accurate, it helps to understand the impact an 18.6% absolute risk reduction in NucST may have.

## LIMITATIONS

The limitations of this study include the retrospective nature of the data collection and outcome analysis as well as it being a single-center study. A prospective multicenter study would be important to evaluate if use of this algorithm could be applied in other EDOU. In addition, the electronic medical record abstractors were not blinded to the study hypothesis. However, the recorded data were specific (which test was done and the results positive or negative, etc.) and were not readily open to interpretation that could contribute to bias. We did not control for the experiences of the emergency physicians. However, as the changes were sustained, it is likely not relevant to the outcomes whether or not the emergency physicians had more or less experience with the algorithm. The 30-day MACE follow-up data was limited in that only the hospital electronic medical record data were used; therefore, no individual follow up was performed (ex. phone calls) and patients presenting to other hospitals with adverse events within 30 days was not captured.

It has been argued that the low rates of 30-day MACE seen in low-risk ED CP patients may not warrant ST in this population^20^,^21^ or that use of only serial contemporary biomarkers could be used to discharge patients from the ED without further index testing.^22^,^23^ Current ACC/AHA guidelines for evaluation of low-risk CP patients recommend stress testing as part of the workup prior to ED or OU discharge or as an outpatient within 72 hours.^11^ In our population of low-risk ED CP patients placed in our EDOU, most reside in a highly litigious county. Therefore, our emergency physicians are much less confident with the option of discharge from the ED. Furthermore, the significant proportion of uninsured patients and/or those without an established primary care physician coupled with the challenge of available transportation and lack of assured availability of outpatient ST within 72 hours also makes this option less attractive.

## CONCLUSION

For EDOU patients requiring CP evaluation with a ST, a simple algorithm favoring Non-NucST options and limiting NucST to a specific subset of patients reduced radiation exposure by almost 50% without a change in 30-day MACE. Emergency physician adherence to the algorithm was excellent.

## Figures and Tables

**Table 1:** Emergency department observation unit utilization patterns of nuclear versus non-nuclear stress tests.

Variable %(n) (95%CI)	Period 1 (before algorithm)	Period 2 (after algorithm)	Period 3 (after algorithm)
Nuclear stress tests	40.7% (n=644) (38.3%, 43.1%)	22.1% (n=363) (20.1%, 24.1%)	22.1% (n=412) (20.3%, 24.0%)
Non-nuclear stress tests	59.3% (n=940) (56.9%, 61.7%)	77.9% (n=1282) (75.9%, 79.9%)	77.9% (n=1455) (76.0%, 79.8%)

**Table 2:** Patient demographics, cardiac co-morbidities and major adverse cardiac events by period.

Variable mean (95% CI) or % (n) (95% CI)	Period 1 (before algorithm)	Period 2 (after algorithm)	Period 3 (after algorithm)
Age	55.3 (53.6–56.7)	55.0 (53.3–56.9)	55.2 (53.6–56.9)
% Male	32.2% (77/239) (26.6%–38.4%)	30.2% (71/235) (24.7%–36.4%)	41.0% (98/239) (35.0%–47.3%)
% Black	72% (162/225) (65.8%–77.5%)	81.4% (184/226) (75.8%–86.0%)	72.3% (167/231) (66.2%–77.7%)
H/o CAD	20.9% (50/239) (16.2%–26.5%)	14.0% (33/236) (10.1%–19.0%)	16.7% (40/239) (12.5%–22.0%)
H/o MI	10% (24/239) (6.8%–14.5%)	11.9% (28/236) (8.3%–16.6%)	12.2% (29/238) (8.6%–17.0%)
H/o PCI	11.3% (27/239) (7.9%–15.9%)	10.6% (25/236) (7.3%–15.2%)	15.5 (37/239) (11.4%–10.6%)
H/o CABG	5.4% (13/239) (3.2%–9.1%)	6.8% (16/236) (4.2%–10.7%)	4.6% (11/239) (2.6%–8.1%)
ACS within 30 days of index visit (%)	0.0% (0/239) (0.0%–1.6%)	0.0% (0/236) (0.0%–1.6%)	0.8% (2/239) (0.2%–3.0%)
PCI within 30 days of index visit (%)	0.8% (2/239) (0.2%–3.0%)	0.0% (0/236) (0.0%–1.6%)	0.8% (2/239) (0.0%–1.6%)
CABG within 30 days of index visit (%)	0% (0/239) (0.0%–1.6%)	0% (0/236) (0.0%–1.6%)	0% (0/239) (0.0%–1.6%)
Death within 30 days of index	0% (0/239) (0.0%–1.6%)	0% (0/236) (0.0%–1.6%)	0% (0/239) (0.0%–1.6%)

*H/o,* history of; *CAD*, coronary artery disease; *MI*, myocardial infarction; *PCI*, percutaneous coronary intervention; *CABG*, coronary artery bypass grafting; *ACS*, acute coronary syndrome
